# *Vibrio cholerae* O1 Isolate with Novel Genetic Background, Thailand–Myanmar

**DOI:** 10.3201/eid1906.120345

**Published:** 2013-06

**Authors:** Kazuhisa Okada, Amonrattana Roobthaisong, Witaya Swaddiwudhipong, Shigeyuki Hamada, Siriporn Chantaroj

**Affiliations:** Thailand-Japan Research Collaboration Center on Emerging and Re-emerging Infections, Nonthaburi, Thailand (K. Okada, A. Roobthaisong, S. Hamada);; Osaka University, Osaka, Japan (K. Okada, S. Hamada);; Mae Sot General Hospital, Tak, Thailand (W. Swaddiwudhipong); and National Institute of Health, Nonthaburi (S. Chantaroj)

**Keywords:** cholera, Thailand, Myanmar, Vibrio cholerae O1, classical, El Tor, bacteria, antimicrobial drugs, antibiotics, pandemic, toxin coregulated pilus, enteric infections

**To the Editor:** *Vibrio cholerae* O1, a causative agent of cholera, was classified into 2 biotypes, classical and El Tor (*1*). However, accumulating evidence suggests that atypical El Tor *V. cholerae*, which possesses traits of both classical and El Tor biotypes, has replaced the seventh pandemic prototypic El Tor *V. cholerae* worldwide in recent years. Cholera outbreaks in Thailand during 2007–2010 were caused by atypical El Tor isolates carrying the classical type cholera toxin gene (*2*). Epidemiologic surveys in a Thailand–Myanmar border area during 2008 yielded more than 500 isolates of *V. cholerae* O1. We identified an isolate that possessed the typical El Tor type cholera toxin gene (genotype 3) and designated it MS6 (later assigned strain number DMST28216). It does not belong to either the seventh pandemic prototypic biotype identified in 1961 or the group of atypical El Tor strains found during 1991–present (*3*).

MS6 was isolated from stool samples from a 26-year-old woman (migrant worker) from Myanmar who had been admitted to Mae Sot General Hospital in Tak Province, Thailand, for 3 days with vomiting, watery diarrhea, nausea, fever, and headache. The illness was considered mild to moderate. Acute gastroenteritis was diagnosed on the basis of the symptoms and laboratory results. The key virulence factors of *V. cholerae* O1 include cholera toxin (CTX), which is responsible for profuse watery diarrhea, and a pilus colonization factor known as toxin-coregulated pilus (TCP). The virulence-related genes (*ctxAB* and *tcpA*) and the phage repressor gene (*rstR*) of MS6 had identical sequences to those of the seventh pandemic prototypic El Tor *V. cholerae* O1 N16961 strain. The isolate was found to be positive for enteric bacteria in the Voges-Proskauer test and resistant to polymyxin B (50 units). We further investigated 2 gene clusters, *Vibrio* seventh pandemic island I (VSP-I) and II (VSP-II), associated with the seventh pandemic strains and absent in classical and pre–seventh pandemic strains (*4*–*7*). The common genes on the VSP-I island in N16961, including VC0175, VC0178, VC0180, VC0181, and VC0183, were detected by PCR (*8*) in MS6 but were lacking in VSP-II; 26.9 kb of VSP-II was originally found in N16961. Moreover, PCR analysis showed that the isolate did not possess the VC2346 gene, a specific marker of the seventh pandemic clone (*5*,*9*). However, we found a VC2346 homolog with 83.9% sequence identity with VC2346 at the nucleotide level and 97% at the amino acid level in the 624-bp region. The coding region of the homolog is considered to be shorter than VC2346 (684 bp) because it contains the stop codon, TAG. This homolog was identified in environmental, classical, or pre–seventh pandemic strains of *V. cholerae* O1 (*5*), including MS6, and in 2740–80 (US Gulf Coast, 1980), 3569–08 (US Gulf Coast, 2008), BX33026 (environmental water in Australia, 1986), RC27 (classical, human isolate in Indonesia, 1991), O395 (classical, human isolate in India, 1965), MAK757 and M66–2 (pre–seventh pandemic, human isolate in Indonesia, 1937), and NCTC 8457 (pre–seventh pandemic, human isolate in Saudi Arabia, 1910), excluding seventh pandemic strains. 

This conservation of the homologue of VC2346 in strains isolated over the course of a century and the geographic distribution of the strains suggest a notable biologic function and a specific marker. In addition, we determined the sequences of 15 housekeeping genes which exhibited sequence variations in toxigenic *V. cholerae* (*10*). The results indicated that by comparison, MS6 is closely related to the US Gulf clones (Figure). However, 2 genes, *malP* and *pepN*, of MS6 are remotely related to them and are novel sequence types, based on results of a BLAST (http://blast.ncbi.nlm.nih.gov/Blast.cgi) search. Antimicrobial susceptibility testing by using the disk diffusion method revealed that MS6 was susceptible to chloramphenicol, ciprofloxacin, gentamicin, sulfamethoxazole/trimethoprim, tetracycline, streptomycin, furazolidone, doxycycline, and norfloxacin, and had intermediate susceptibility to ampicillin and erythromycin, suggesting that MS6 had not been exposed to several antimicrobial drugs. The *V. cholerae* SXT element, which usually shows code drug-resistance markers, integrates into a specific site of the *prfC* gene. In MS6, the complete *prfC* gene was detected. The accession numbers of DDBJ for nucleotide sequences determined in this study are AB699244-AB699265. MS6 possesses unique properties in terms of the ribotype, pulsed-field gel electrophoresis pattern, and multiple-locus variable-number tandem-repeat analysis profile, compared with other *V. cholerae* O1 isolates (*2*).

**Figure:**
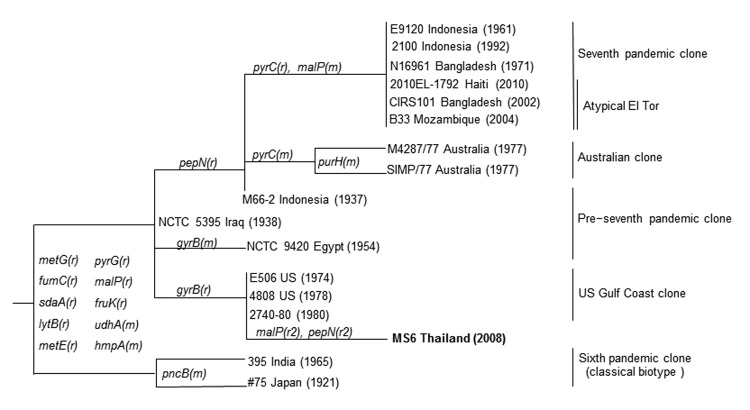
Relationships among MS6, *Vibrio cholerae* O1 strain, isolated in Thailand in 2008, and other *V. cholerae* O1 strains based on 15 housekeeping genes referenced in Salim et al. (*10*). **Boldface** indicates the MS6 strain. The mutational (m) and recombinational (r) changes with gene names are marked on the branches (r ≠ r2). Numbers in parentheses represent the year of isolation. DNA gyrase subunit B gene (g*yrB*) of MS6 is 22 nt differences from that of the seventh pandemic clone. Two genes of MS6, *malP* and *pepN*, exhibit novel sequence types based on results of a BLAST search (http://blast.ncbi.nlm.nih.gov/Blast.cgi).

This case was probably an episode of sporadic cholera from indigenous *V. cholerae* O1, such as US Gulf Coast and Australian clones, which are mainly associated with environmental sources. We have been unable to isolate another MS6-like clone, which could have escaped detection because of low prevalence or might exist in a dormant state in a rural area. Nevertheless, the transmission route and its pathogenicity must be of concern for public health.

## References

[1] Sack DA, Sack RB, Nair GB, Siddique AK. Cholera. Lancet. 2004;363:223–33. 10.1016/S0140-6736(03)15328-7 14738797

[2] Okada K, Roobthaisong A, Nakagawa I, Hamada S, Chantaroj S. Genotypic and PFGE/MLVA analyses of *Vibrio cholerae* O1: geographical spread and temporal changes of isolates during the 2007–2010 cholera outbreaks in Thailand. PLoS ONE. 2012;7:e30863. 10.1371/journal.pone.0030863 22292065 PMC3265523

[3] Safa A, Nair GB, Kong RYC. Evolution of new variants of *Vibrio cholerae* O1. Trends Microbiol. 2010;18:46–54. 10.1016/j.tim.2009.10.003 19942436

[4] Dziejman M, Balon E, Boyd D, Fraser CM, Heidelberg JF, Mekalanos JJ. Comparative genomic analysis of *Vibrio cholerae*: genes that correlate with cholera endemic and pandemic disease. Proc Natl Acad Sci U S A. 2002;99:1556–61. 10.1073/pnas.042667999 11818571 PMC122229

[5] Chun J, Grim CJ, Hasan NA, Lee JH, Choi SY, Haley BJ, Comparative genomics reveals mechanism for short-term and long-term clonal transitions in pandemic *Vibrio cholerae.* Proc Natl Acad Sci U S A. 2009;106:15442–7. 10.1073/pnas.0907787106 19720995 PMC2741270

[6] Taviani E, Grim CJ, Choi J, Chun J, Haley B, Hasan NA, Discovery of novel *Vibrio cholerae* VSP-II genomic islands using comparative genomic analysis. FEMS Microbiol Lett. 2010;308:130–7. 20528940 10.1111/j.1574-6968.2010.02008.x PMC2925232

[7] Grim CJ, Choi J, Chun J, Jeon YS, Taviani E, Hasan NA, Occurrence of the *Vibrio cholerae* seventh pandemic VSP-I island and a new variant. OMICS. 2010;14:1–7. 10.1089/omi.2009.0087 20141327

[8] O’Shea YA, Reen FJ, Quirke AM, Boyd EF. Evolutionary genetic analysis of the emergence of epidemic *Vibrio cholerae* isolates on the basis of comparative nucleotide sequence analysis and Multilocus Virulence Gene Profiles. J Clin Microbiol. 2004;42:4657–71 . 10.1128/JCM.42.10.4657-4671.2004 15472325 PMC522369

[9] Talkington D, Bopp C, Tarr C, Parsons MB, Dahourou G, Freeman M, Characterization of toxigenic *Vibrio cholerae* from Haiti, 2010–2011. Emerg Infect Dis. 2011;17:2122–9. 22099116 10.3201/eid1711.110805 PMC3310580

[10] Salim A, Lan R, Reeves PR. *Vibrio cholerae* pathogenic clones. Emerg Infect Dis. 2005;11:1758–60 . 10.3201/eid1111.041170 16318732 PMC3367346

